# A necroptosis related prognostic model of pancreatic cancer based on single cell sequencing analysis and transcriptome analysis

**DOI:** 10.3389/fimmu.2022.1022420

**Published:** 2022-10-07

**Authors:** Liang Chen, Xueming Zhang, Qixiang Zhang, Tao Zhang, Jiaheng Xie, Wei Wei, Ying Wang, Hongzhu Yu, Hongkun Zhou

**Affiliations:** ^1^ Department of Hepatobiliary and Pancreatic Surgery, Conversion Therapy Center for Hepatobiliary and Pancreatic Tumors, First Hospital of Jiaxing, Affiliated Hospital of Jiaxing University, Jiaxing, Zhejiang, China; ^2^ Department of Neurosurgery, First Affiliated Hospital of Nanjing Medical University, Nanjing, China; ^3^ Department of General Surgery, Fuyang Hospital Affiliated to Anhui Medical University, Fuyang, China; ^4^ Department of Burn and Plastic Surgery, The First Affiliated Hospital of Nanjing Medical University, Nanjing, China; ^5^ Department of Anesthesiology, Jiaxing First Hospital, Jiaxing, China; ^6^ Department of Neurosurgery, Children's Hospital of Nanjing Medical University, Nanjing, China

**Keywords:** pancreatic cancer, necroptosis, programmed cell death, prognostic model, single-cell sequencing analysis, bioinformatics

## Abstract

**Background:**

As a tumor type with high mortality and poor therapeutic effect, the pathogenesis of pancreatic cancer is still unclear. It is necessary to explore the significance of necroptosis in pancreatic cancer.

**Methods:**

Pancreatic cancer transcriptome data were obtained from the TCGA database, ICGC database, and GSE85916 in the GEO database. The TCGA cohort was set as a training cohort, while the ICGC and GSE85916 cohort were set as the validation cohorts. Single-cell sequencing data of pancreatic cancer were obtained from GSE154778 in the GEO database. The genes most associated with necroptosis were identified by weighted co-expression network analysis and single-cell sequencing analysis. COX regression and Lasso regression were performed for these genes, and the prognostic model was established. By calculating risk scores, pancreatic cancer patients could be divided into NCPTS_high and NCPTS_low groups, and survival analysis, immune infiltration analysis, and mutation analysis between groups were performed. Cell experiments including gene knockdown, CCK-8 assay, clone formation assay, transwell assay and wound healing assay were conducted to explore the role of the key gene EPS8 in pancreatic cancer. PCR assays on clinical samples were further used to verify EPS8 expression.

**Results:**

We constructed the necroptosis-related signature in pancreatic cancer using single-cell sequencing analysis and transcriptome analysis. The calculation formula of risk score was as follows: NCPTS = POLR3GL * (-0.404) + COL17A1 * (0.092) + DDIT4 * (0.007) + PDE4C * (0.057) + CLDN1 * 0.075 + HMGA2 * 0.056 + CENPF * 0.198 +EPS8 * 0.219. Through this signature, pancreatic cancer patients with different cohorts can be divided into NCPTS_high and NCPTS_low group, and the NCPTS_high group has a significantly poorer prognosis. Moreover, there were significant differences in immune infiltration level and mutation level between the two groups. Cell assays showed that in CAPAN-1 and PANC-1 cell lines, EPS8 knockdown significantly reduced the viability, clonogenesis, migration and invasion of pancreatic cancer cells. Clinical PCR assay of EPS8 expression showed that EPS8 expression was significantly up-regulated in pancreatic cancer (*P<0.05).

**Conclusion:**

Our study can provide a reference for the diagnosis, treatment and prognosis assessment of pancreatic cancer.

## Introduction

Pancreatic cancer is one of the most deadly cancers worldwide and is characterized by rapid growth and invasion (1, 2). Ductal adenocarcinoma is the most common pathologic type of pancreatic cancer (3). Due to the inobvious early symptoms of pancreatic cancer, such as loss of appetite, abdominal pain, back pain, many pancreatic cancer patients have often delayed diagnosis, which leads to a significant number of patients being already diagnosed at an advanced stage, often accompanied by distant metastasis (4–6). Currently, treatment for pancreatic cancer is tricky (7). Although a combination of surgery, adjuvant therapy, neoadjuvant therapy, and immunotherapy has been widely used in patients with pancreatic cancer, only 20% of patients are effective (7–10). Postoperative recurrence, recurrent drug resistance, and persistent low response to treatment are still problems in the treatment of pancreatic cancer (11). The complex tumor microenvironment of pancreatic cancer may be a key factor leading to these adverse outcomes (12). Therefore, it is necessary to explore the changes of the tumor microenvironment in pancreatic cancer to provide ideas for the diagnosis and treatment of pancreatic cancer.

Necroptosis is a newly defined type of programmed cell death, which is distinctly different from necrosis (13). Necroptosis is a regulated type of cell death (14). However, necrosis is a passive process of cell death in response to drastic changes in the external environment (15). The role of necroptosis in cancer has been tentatively discussed (16). However, necroptosis's role in cancer is unclear. On the one hand, some studies have suggested that necroptosis is a cancer suppressor and that inducing necroptosis in cancer cells can reverse their resistance to cell death (17). On the other hand, necroptosis has been implicated in some studies as a cancer promoter (17). The immune active substances and reactive oxygen species released in this process promote the activation of many cancer pathways, participate in the regulation of the immune microenvironment, and promote the proliferation and invasion of tumor cells (18). It's time to explore the role of necroptosis genes in pancreatic cancer.

Here, we conducted comprehensive bioinformatics analysis, including single-cell sequencing analysis, expression analysis, survival analysis, immune microenvironment analysis, and mutation analysis. From these analyses, we constructed a necroptosis-associated prognostic signature in pancreatic cancer. According to the signature, pancreatic cancer patients can be divided into high-risk and low-risk groups. Survival analysis based on risk grouping is a good method to evaluate the prognosis of patients with pancreatic cancer. The analysis of the immune microenvironment can provide a reference for understanding the immune mechanism of pancreatic cancer. Overall, our study may provide new insights into the diagnosis and treatment of pancreatic cancer.

## Methods

### Transcriptome data download and processing

The transcriptome data used in this study came from TCGA, ICGC and GEO databases. TCGA database collects transcriptome data, mutation data and clinical data of various cancer types, which greatly facilitates cancer research. In this study, pancreatic cancer transcriptome data and clinical data from the TCGA database were downloaded as a training cohort. The ICGC database also contains transcriptome data and clinical data for various types of cancer, which are often used to validate the results of TCGA data analysis. In this study, data from the ICGC database on pancreatic cancer PA-AU cohort and PA-CA cohort were downloaded as validation cohort. Meanwhile, the pancreatic cancer dataset GSE85916 from GEO database was also downloaded as a validation cohort. All transcriptome data were transformed by log2 for subsequent analysis.

### Single cell sequencing data download and processing

GEO database contains a large number of single cell sequencing data. In this study, a single cell sequencing dataset of pancreatic cancer was obtained through GEO database, which contained 15 samples in total. Firstly, the cells and genes included in the study were filtered according to the following criteria: 1) cells expressing less than 200 genes were removed; 2) genes expressed in less than 3 cells were removed. 3) Cells whose number of expressed genes fluctuated between 200 and 7000 were retained. 4) Cells whose percentage of mitochondrial genes was less than 10% were retained. SCTransform function was used to remove the influence of cell cycle on subsequent results. The standardized method of "SCT" is used to integrate different samples and remove batch effect. The number of selected dimensions was set as 20, and KNN method was used for dimension reduction and clustering analysis. Then the cells were annotated by singleR package and marker genes of the cells.

### Sources of necroptosis-related genes

GeneCard database integrates numerous literatures and contains a great deal of information related to gene function. In this study, genes related to necroptosis were downloaded from this database, and then sequenced according to the degree of correlation from high to low, and genes with correlation greater than 0.2 were retained.

### ssGSEA analysis

ssGSEA analysis is commonly used to calculate the enrichment fraction of a particular gene set in each sample, which represents the absolute enrichment degree of the gene set in each sample. In this study, ssGSEA analysis was used to calculate the enrichment fraction of necroptosis in each sample.

### Weighted gene co-expression network analysis

Weighted gene co-expression network analysis (WGCNA) is a systems biological method used to describe gene association patterns between different samples. It can be used to identify highly covarying gene sets and to identify candidate biomarker genes based on the interconnectedness of gene sets and the association between gene sets and phenotypes. In this study, this analysis was used to identify a gene set closely associated with necroptosis in pancreatic cancer.

### Construction and evaluation of the prognostic model

Univariate COX analysis was first performed to identify necroptosis genes associated with prognosis (P <0.05). Subsequently, LASSO regression and tenfold cross-validation were used to further identify the key genes affecting patient outcomes. Finally, prognostic models are constructed based on these genes and their coefficients. Patients in all cohorts were divided into high and low NCPTS groups based on median NCPTS values. Survival analysis was performed for both groups and the accuracy of the model was evaluated.

### External validation of the prognostic model

PA-AU cohort, PA-CA cohort and GSE85916 cohort were used to further evaluate and validate the model. Patients were divided into two groups based on median NCPTS value and survival analysis was performed. In addition, the model's accuracy, independent prognostic value, and ability to distinguish between patients were assessed.

### Analysis of immune infiltration and immunotherapy

The "ESTIMATE" package was used to calculate immune and stromal scores. The "ImmuneSubtypeClassifier" package was used to calculate the immune subtypes for each sample. The TIMER 2.0 web site was used to analyze the immune infiltration results of samples from the TCGA database. The TIDE database was used to calculate TIDE scores for each sample to assess the efficacy of immunotherapy.

### Mutation landscape analysis

The "MAfTools" package was used to download pancreatic cancer mutation data from the TCGA database, selecting the mutation data type as "MutecT2". The first 20 mutated genes from the different groups were then shown. The function TMB of the "MAfTools" package was used to calculate the tumor mutation load (TMB) for each sample.

### Gene set enrichment analysis

GSEA is a common calculation used to assess whether there is a statistically significant difference between two biological data sets in a preset of genes. In this study, this analysis was used to calculate the major activated pathways in the high NCPTS risk group.

### Construction of the nomogram

The Nomogram can be used to visualize the results of Cox or Logistic regression. Moreover, it establishes scoring criteria by the size of regression coefficients of all independent variables, and each patient can be calculated to obtain an overall score to evaluate the incidence of patient outcome events. In this study, the 1, 3 and 5 mortality of this patient was predicted by combining clinical data and model values of sample TCGA-2J-AABK.

### Cell lines, culture conditions and cell transfection

Shanghai Institutes for Biological Sciences provided Capan-1 and PANC-1 cells (Shanghai, China). These cells were grown in DMEM supplemented with 10% fetal bovine serum (FBS) and 1% penicillin-streptomycin solution. All of the cells were grown at 37°C with 5% CO2. Lipofactamine3000 (Thermo Fisher Scientific, Waltham, MA, USA) was used to transfect cells with siRNAs (RiboBio, Guangzhou, China) according to the manufacturer's recommendations. 5′- GCCAAACUGAAGUCUCAUAUUTT-3′ (siEPS8-1), 5′- CCAACUUCUAAUCGCCAUAUATT-3′ (siEPS8-2), and 5’-GCUAGUGAUUCAGGAGUGGAATT-3’ (siEPS8-3) were the three target sequences for siRNA for EPS8.

### Quantitative real−time polymerase chain reaction

Six pancreatic cancer patients were recruited from May 2021 to March 2022 at Fuyang Hospital Affiliated to Anhui Medical University. The study was approved by the Ethics Committee of Fuyang Hospital Affiliated to Anhui Medical University (No.KY2022010). The tumor tissues and para-tumor tissues were obtained during the operation for PCR assay. TRIzol reagent (Invitrogen, CA, USA) was used to extract total RNA from cell lines according to the manufacturer's instructions. The PrimeScript RT Reagent Kit was used to make cDNA (Takara, Nanjing, China). On an ABI Stepone plus PCR equipment, qRT-PCR was performed using AceQ Universal SYBR qPCR Master Mix (Vazyme, Nanjing, China) (Applied Biosystems, FosterCity, CA, USA). The following were the primers utilized in this study: EPS8(Forward): TGAATGGCTACGGATCATCACC; EPS8(Reverse):CACTGTCCCGTGCATAATTCT. ACTB(Forward):GTCATTCCAAATATGAGATGCGT;ACTB(Reverse):GCATTACATAATTTACACGAAAGCA. Relative quantification was determined using the 2^-ΔΔCt^ method.

### CCK-8 assay, Colony formation analysis, migration and invasion assays, and wound healing

These experimental methods have been reported in our previous studies.

## Results

**Figure 1:**
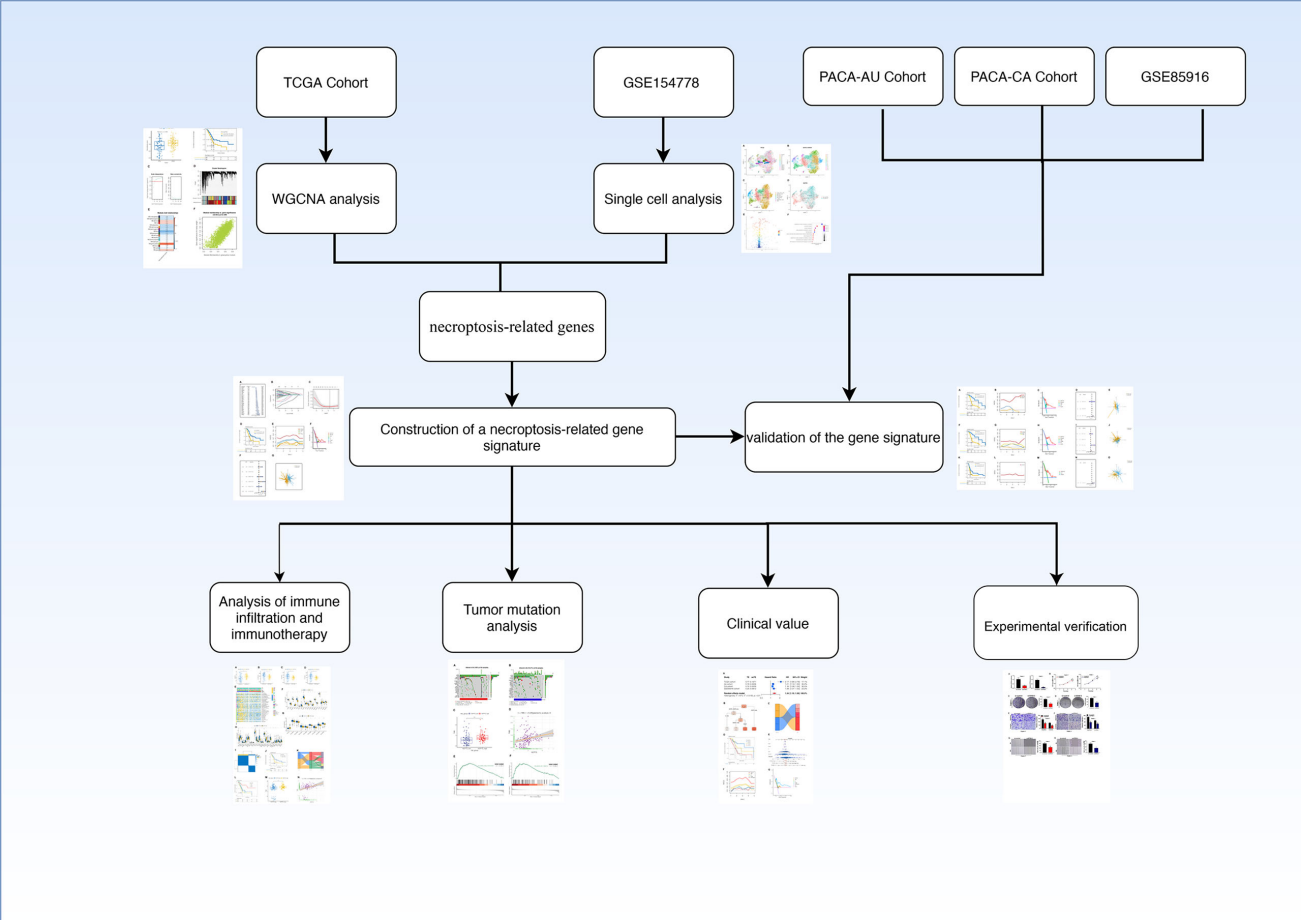
Flow chart.

The flow chart of this study was shown in **Figure 1**.

### Weighted gene co-expression network analysis

**Figure 2:**
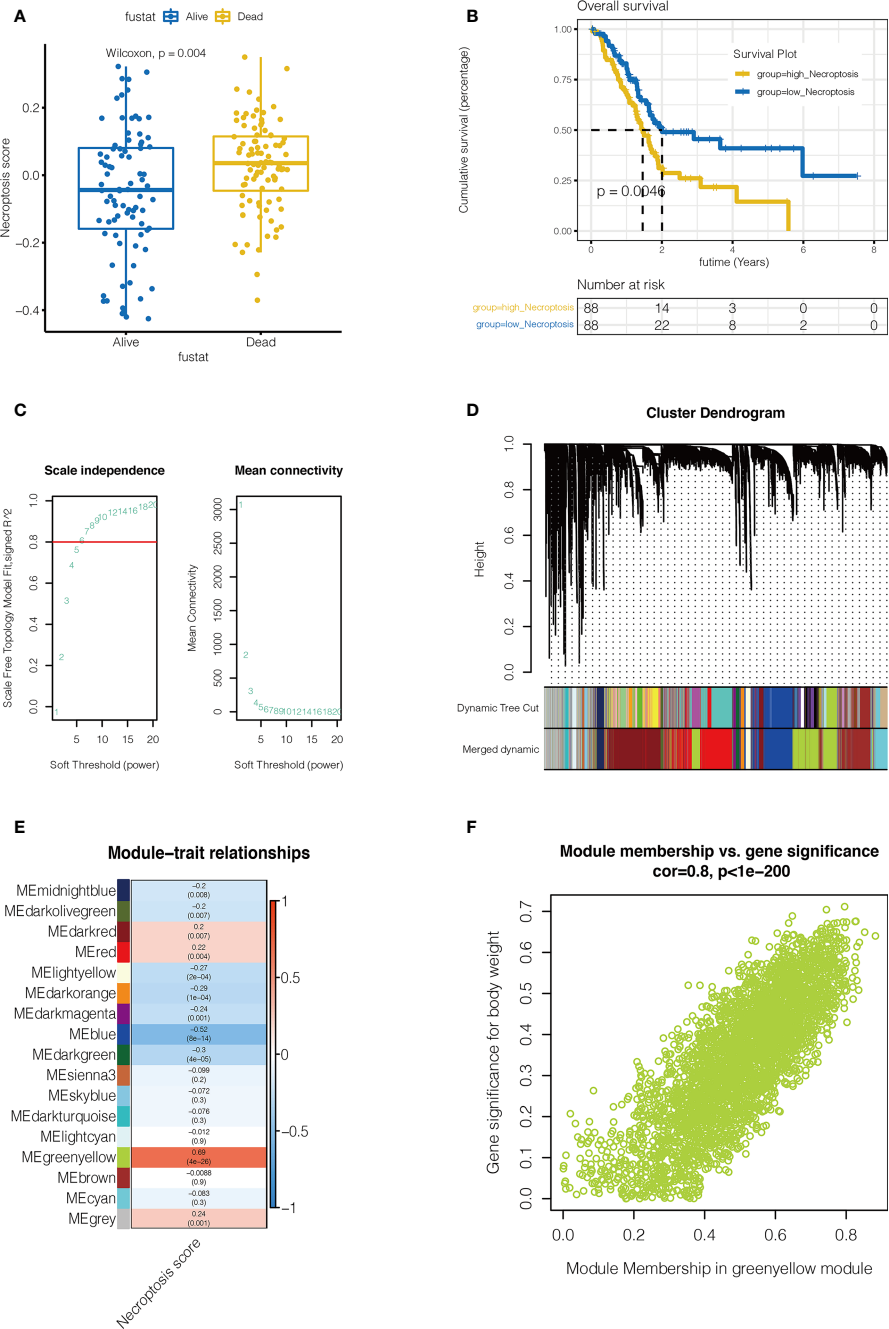
Single sample gene set enrichment analysis(ssGSEA) and Weighted Gene Co-expression Network Analysis (WGCNA). **(A, B)** ssGSEA. The Necroptosis score for each pancreatic cancer patient in the TCGA database was calculated. Necroptosis scores were higher in pancreatic cancer patients who died (P=0.004). Pancreatic cancer patients in the high-necroptosis group had worse outcomes (P=0.0046). **(C)** WGCNA. When the soft domain value is 7, R^2>0.8, the data is more consistent with the power-law distribution, and mean connectivity tends to be stable, which is suitable for subsequent analysis. **(D)** First the minimum number of modules is set to 100, deepSplit is 3, and a total of 27 non-grey modules are obtained. Then, the similarity domain value of modules was set to 0.4, and the modules lower than this value were combined to obtain 16 non-gray modules. **(E)** MEgreenyellow module was most closely associated with necroptosis, containing 3352 genes (COR = 0.8, P <0.001). **(F)** The correlation between gene significance for body weight and module membership (COR = 0.8, P <0.001).

As shown in **Figures 2A, B**, necroptosis score was calculated by ssGSEA for each sample. Patients were divided into high-necroptosis and low-necroptosis groups according to the median necroptosis score, and higher necroptosis scores were found among pancreatic cancer patients who died. Furthermore, the prognosis of patients in the high-necroptosis group was poor (P <0.05), suggesting that necroptosis is a risk factor for pancreatic cancer. WGCNA was performed to further search for gene sets that were covarying with necroptosis. As shown in **Figure 2C**, when the soft domain value is 7, R^2>0.8, the data is more consistent with the power-law distribution, and mean connectivity tends to be stable, which is suitable for subsequent analysis. As shown in **Figure 2D**, the minimum number of modules were set to 100 and deepSplit to 3 and a total of 27 non-grey modules are obtained. Then, the similarity domain value of modules was set to 0.4, and the modules lower than this value were merged, and finally 16 non-gray modules were obtained. We found that, as shown in **Figures 2E, F**, MEgreenyellow module was most closely associated with necroptosis, containing 3352 genes (COR = 0.8, P <0.001).

### Single-cell sequencing analysis

**Figure 3:**
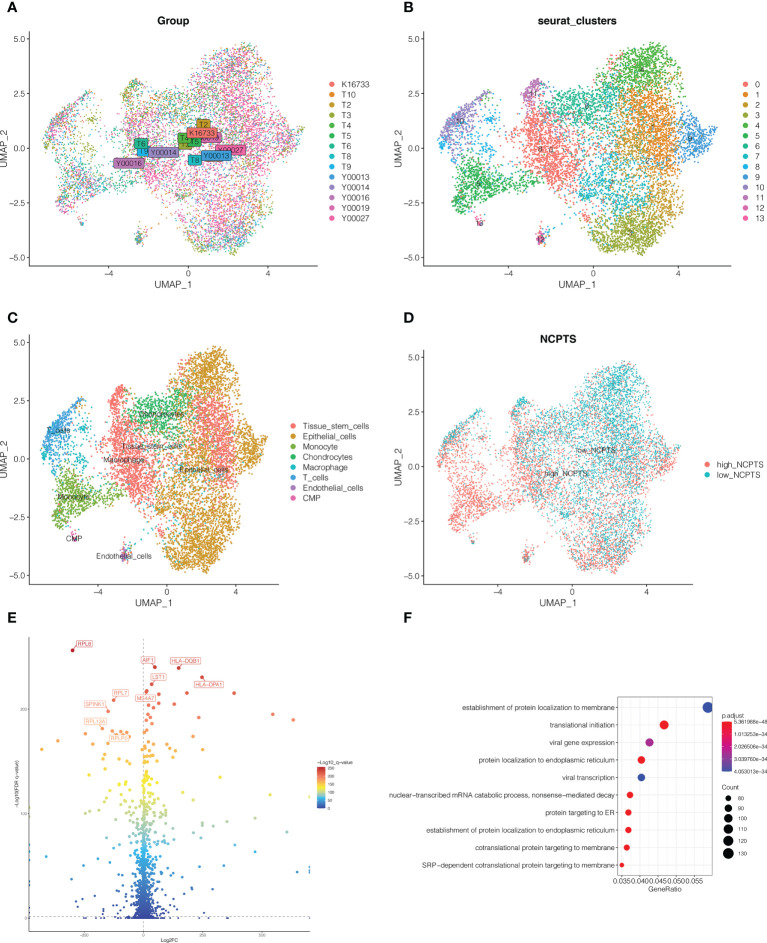
Single cell sequencing analysis. **(A)** A total of 14 samples were included in the study. Cells were evenly distributed among all samples, suggesting no obvious batch effect among all samples. **(B)** Dimension reduction and cluster analysis. All cells were clustered into 14 clusters. **(C)** Classification of cells. A total of 8 cell types can be found, such as epithelial cell, macrophage cell and T cell. **(D)** The cells were divided into high and low groups, namely high-NCPTs and low-NCPTs. **(E)** Differential expression gene analysis between high-NCPTs group and low-NCPTs group. A total of 2518 differentially expressed genes between these two groups were obtained(P<0.05). The intersection of these differentially expressed genes with the genes of MEgreenyellow module in WGCNA was obtained to obtain 805 most related genes. **(F)** Gene ontology(GO) enrichment analysis of differentially expressed genes.

As shown in **Figure 3A**, 14 samples were included in the study, and the cell distribution among each sample was relatively uniform, indicating that there was no obvious batch effect among samples, which could be used for subsequent analysis. Subsequently, all cells were clustered into 14 clusters (**Figure 3B**). According to the genetic characteristics of each cluster, different cell types were annotated through the singleR package. As shown in **Figure 3C**, a total of 8 cell types can be found, such as epithelial cell, macrophage cell and T cell. The percentage of necroptosis genes per cell was then determined based on the characteristics of necroptosis genes in each cell. According to the median value, the cells were divided into high and low groups, namely high-NCPTs and low-NCPTs (**Figure 3D**). Then, 2518 differentially expressed genes between these two groups were obtained by difference analysis with P <0.05. As shown in **Figure 3E**, the top 10 genes with the most significant changes were RPL8, AIF1,HLA-DQB1, LST1, HLA-DPA1, RPL7, MS4A7, SPINK1, RPL13A, and RPLP0. At the same time, gene ontology enrichment analysis indicated that these genes were mainly related to mRNA metabolism and protein localization (**Figure 3F**). Finally, 805 genes closely related to necroptosis were obtained by intersection of differentially expressed genes and genes of MEgreenyellow module.

### Construction and evaluation of the prognostic model in the TCGA cohort

**Figure 4:**
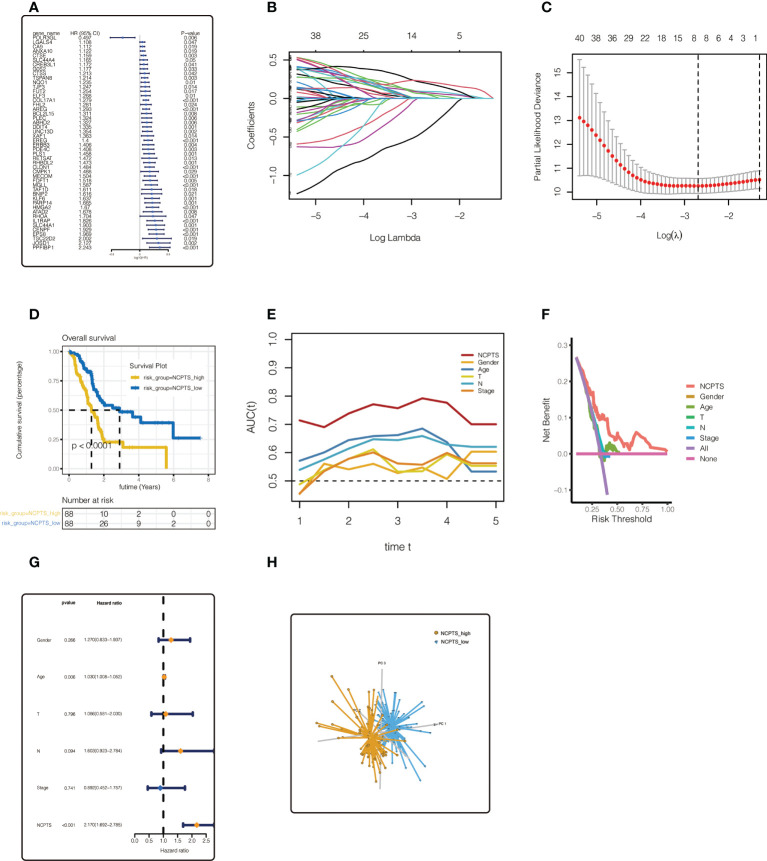
Establishment of the prognostic model in TCGA cohort. **(A)** Univariate Cox analysis of 805 most related genes. 48 necroptosis genes were identified by univariate Cox analysis of 805 necroptosis genes in TCGA and PA-AU cohort. **(B, C)** LASSO regression analysis. The model constructed by 8 genes is finally obtained. **(D)** Survival anaylsis. Patients with NCPTS_high had a poor prognosis (P <0.001). **(E)** ROC curve analysis. The AUC of model value NCPTS was maintained at about 0.75. **(F)** Decision curve analysis. **(G)** Multivariate Cox analysis. NCPTS was an independent prognostic factor for pancreatic cancer patients. **(H)** Principal component analysis (PCA). The model can distinguish patients into NCPTS_high and NCPTS_low groups well.

To further identify necroptosis genes associated with prognosis, 48 necroptosis genes were identified by univariate Cox analysis of 805 necroptosis genes in TCGA and PA-AU cohorts with p<0.05. In TCGA cohort, forest map was used to show the univariate Cox analysis results of the 48 genes (**Figure 4A**). LASSO regression was then performed. As shown in **Figures 4B, C**, the optimal Lambda is 0.0678, and the model constructed by 8 genes is finally obtained. Model value NCPTS = POLR3GL * (-0.404) + COL17A1 * (0.092) + DDIT4 * (0.007) + PDE4C * (0.057) + CLDN1 * 0.075 + HMGA2 * 0.056 + CENPF * 0.198 +EPS8 * 0.219. Patients were then divided into NCPTS_high and NCPTS_low groups according to the median value. As shown in **Figure 4D**, patients in the NCPTS_high group had a poor prognosis (P <0.001). ROC curve found that the AUC of model value NCPTS in predicting patient prognosis was maintained at about 0.75, which was significantly better than clinical characteristics, such as gender, age, stage, etc. (**Figure 4E**). Similarly, decision curve analysis found that patients benefited most from clinical intervention based on NCPTS compared to clinical characteristics (**Figure 4F**). Multivariate Cox analysis showed that NCPTS was an independent prognostic factor for pancreatic cancer patients (**Figure 4G**). In addition, the model can better distinguish patients into NCPTS_high and NCPTS_low groups (**Figure 4H**).

### External validation and evaluation of the prognostic model

**Figure 5:**
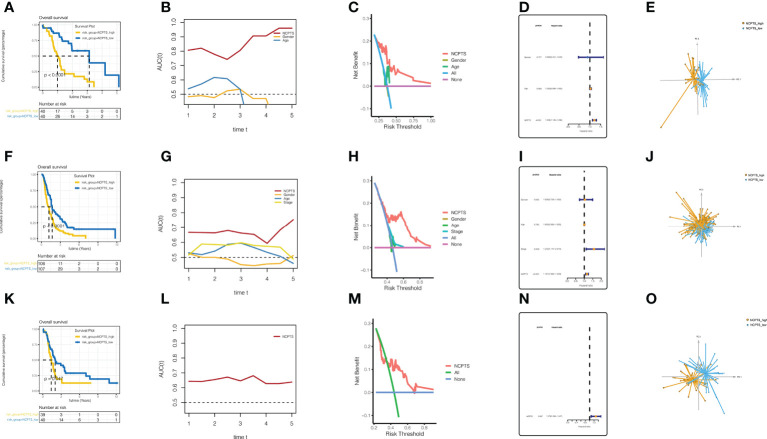
External validation and evaluation of the prognostic model. **(A–E)** Validation and evaluation of the model in the PA-AU cohort. **(F–J)** Validation and evaluation of the model in the PA-CA cohort. **(K–O)** Validation and evaluation of the model in the GSE85916 cohort.

Similar results were observed in the external validation queues PA-AU (**Figures 5A–E**), PA-CA (**Figures 5F–J**), and GSE85916 (**Figures 5K–O**). In the PA-AU cohort (**Figures 5A–E**) and PA-CA cohort (**Figures 5F–J**), patients in the NCPTS_high group had poorer outcomes than those in the NCPTS_low group. Moreover, ROC analysis of prognosis showed that AUC under the curve fluctuated between 0.85 and 0.7, which had a better effect on prognosis assessment of patients, and was better than clinical characteristics such as gender and age (**Figures 5B, G**). Decision curve analysis also suggested that patients benefit most from interventions based on NCPTS (**Figures 5C, H**). Multivariate Cox analysis suggested that NCPTS was an independent factor affecting the prognosis of patients (**Figures 5D, I**), and the model could well distinguish patients into NCPTS_high and NCPTS_low groups (**Figures 5E, J**). Similarly, in the GSE85916 cohort, it was found that the prognosis of patients with NCPTS_high was poor, and the AUC under the curve was stable according to the prognostic ROC analysis, with a fluctuation of 0.65 (**Figures 5K, L**). The decision curve analysis also suggested that clinical intervention based on NCPTS had a reasonable benefit effect (**Figure 5M**). Univariate Cox analysis showed that NCPTS was a prognostic factor (**Figure 5N**). Moreover, the model can still distinguish patients into NCPTS_high and NCPTS_low groups (**Figure 5O**).

### Immune infiltration analysis and identification of patients more suitable for immunotherapy

**Figure 6:**
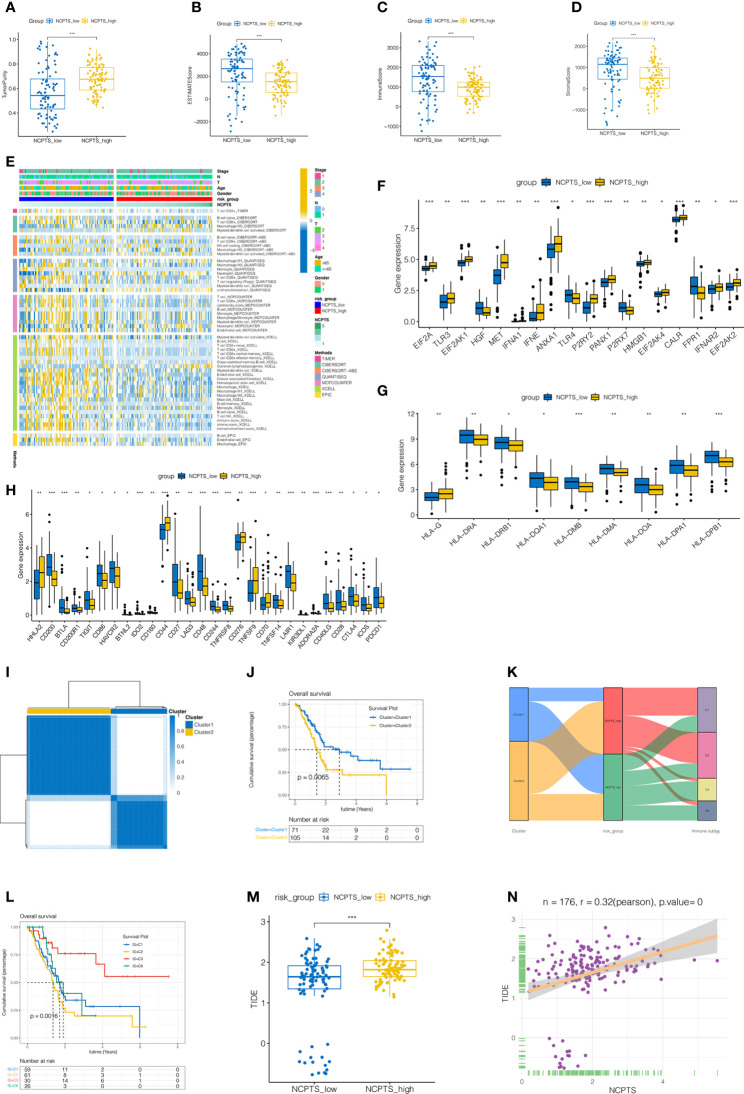
Immune infiltration analysis and identification of patients more suitable for immunotherapy in TCGA cohort. **(A–D)** Differences in immune microenvironment scores among different NCPS groups. Compared with NCPTS_high group, NCPTS_low group had higher Estimate Score, Immune Score, and Stromal Score (P<0.001), but the tumor purity was lower in the NCPTS_low group. **(E)** Immue landscape of different NCPS groups. **(F)** Differences in expression of immunogenic cell death (ICD) genes between the two groups. **(G)** Differences in expression of human leukocyte antigen (HLA) gene genes between the two groups. **(H)** Differences in expression of immune checkpoint (ICP) gene genes between the two groups. **(I)** Unsupervised consistency cluster analysis. Patients can be divided into two clusters according to the expression of model genes. **(J)** Survival analysis showed a worse prognosis for cluster2(P=0.0065). **(K)** Sankey diagram shows the relationship among cluster types, risk groups and immune subtypes. **(L)** Survival analysis of different immune subtypes. **(M)** TIDE scores in different NCPS risk groups. TIDE scores in different NCPS risk groups. TIDE score was higher in NCPS_high group. ***P<0.001 **(N)** Pearson correlation analysis showed a positive correlation between NCPTS and TIDE. *P<0.05; **P<0.01;***P<0.001.

The immune microenvironment plays an important role in the prognosis of patients. Immune infiltration between NCPTS_low and NCPTS_high groups was analyzed. As shown in **Figures 6A–D**, compared with the NCPTS_high group, the Estimate score, Immune score and Stromal score were higher in the NCPTS_low group (P<0.001), but the tumor purity was lower in the NCPTS_low group. Similar to the above results, we found higher levels of immune cell infiltration, such as T cells and B cells, in the NCPTS_low group, as shown in **Figure 6E**. Immunogenic cell death genes (ICDs), human leukocyte antigens (HLAs) and immune checkpoints (ICPs) are also closely related to the occurrence and development of tumors. As shown in **Figure 6F**, in the NCPTS_low group, the expression of ICDs showed an overall downward trend, but the expressions of HGF, TLR4, P2RX7 and FPR1 were up-regulated. As shown in **Figure 6G**, in the NCPTS_low group, the overall expression of HLAs was up-regulated, but the expression of HLA-G was down-regulated. As shown in **Figure 6H**, in the NCPTS_low group, the expression of ICPs was generally up-regulated, such as PDCD1, CD27, CTLA4, etc. Then, unsupervised clustering analysis was performed on the patients according to the genes in the model, and it was found that the patients could be clustered into 2 Clusters (**Figures 6I–J**). Compared with Cluster1, Cluster2 had a worse prognosis (P<0.05). Subsequently, the relationship between NCPTS grouping and Clusters as well as immune subtypes was explored, as shown in **Figure 6K**. Cluster1 was found to be mainly related to the NCPTS_low group, and immune subtypes C3 and C6 were mainly distributed in the NCPTS_low group. Cluster2 was mainly associated with NCPTS_high group, and immune subtypes C1 and C2 were mainly distributed in NCPTS_high group. Subsequently, survival analyses between immune subtypes were performed. As shown in **Figure 6L**, we found that the prognosis of immune subtype C3 group was the best, while C3 was mainly distributed in the NCPTS_low group, which was consistent with the previous results. In conclusion, we found that NCPTS_low group had a higher level of immune cell infiltration, and the expression of ICPs and HLAs was also higher, and the immune subtype C2 was mainly distributed in this group. We hypothesized that the NCPTS_low group might be immune "hot" tumors, while the NCPTS_HIGH group might be immune "cold" tumors. Then the immunotherapy response between the NCPTS groups was explored as shown in **Figures 6M**, N. It was found that TIDE was lower in the NCPTS_low group, suggesting a lower possibility of immune escape from tumors and a greater possibility of immunotherapy benefit for patients (**Figure 6M**). Moreover, TIDE and NCPTS showed a strong positive correlation (**Figure 6N**), that is, with the increase of NCPTS, TIDE value also increased, the probability of tumor immune escape also increased, and the benefit of immunotherapy was less.

### Mutation landscape analysis

**Figure 7:**
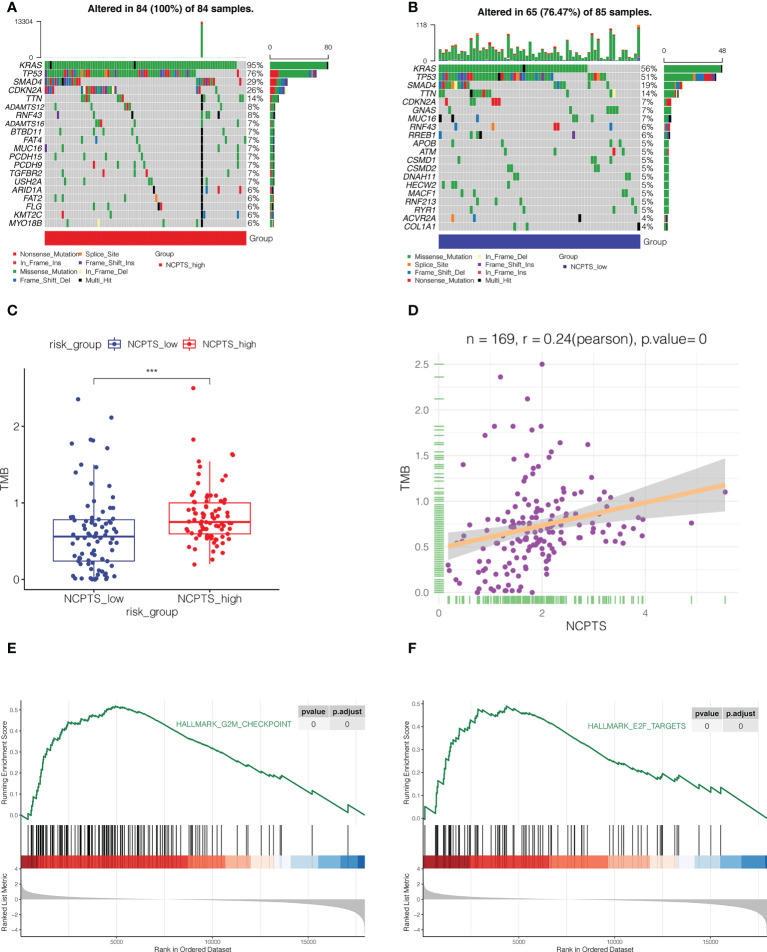
Mutation landscape analysis. **(A)** The mutation landscape of NCPTS_high group. **(B)** The mutation landscape of NCPTS_low group. **(C)** Analysis of tumor mutation load(TMB) between NCPTS_high and NCPTS_low groups. The TMB of the NCPTS_high group was higher. ***P<0.001 **(D)** Pearson correlation analysis showed a positive correlation between NCPTS and TMB. **(E, F)** GSEA showed that G2M CHECKPOINT and E2F TARGETS pathways related to cell cycle were activated in the NCPTS_high group.

Gene mutation also plays an important role in the development of tumor and prognosis of patients. As shown in **Figures 7A, B**, the top 5 most frequently mutated genes in both the NCPTS_high group and the NCPTS_low group were KRAS, TP53, SMAD4, CDKN2A and TTN. TMB differences between the two groups were then analyzed. As shown in **Figures 7C, D**, TMB in the NCPTS_high group was higher, and TMB was positively correlated with NCPTS (P<0.001). TMB is often associated with poor patient prognosis, which may be the reason for the poor prognosis in the NCPTS_high group. Subsequently, enrichment analysis of pathways between the two groups showed that G2M_CHECKPOINT and E2F_TARGETS pathways related to cell cycle were activated in the NCPTS_high group (**Figures 7E, F**).

### Clinical significance of the model

**Figure 8:**
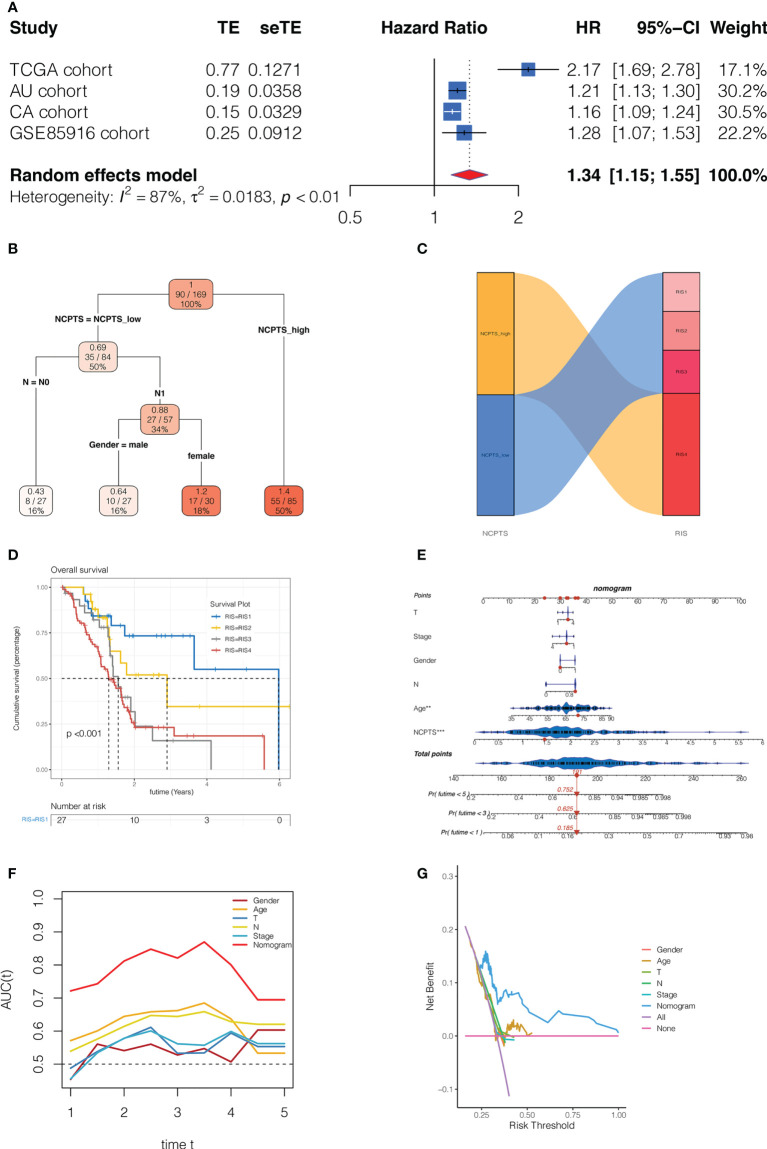
Clinical significance of the model. **(A)** Meta-analysis of the prognostic impact of NCPTS in different cohorts. Pooled HR values were found to show that NCPTS remained a contributing factor to poor patient outcome. **(B)** Decision tree analysis. Patients can be divided into 4 risk groups RIS1, RIS2, RIS3 and RIS4 according to the level of NCPTS, gender and N stage. **(C)** RIS4 patients were all distributed in the NCPTS_high group, while RIS1, RIS2, and RIS3 were distributed in the NCPTS_low group. **(D)**Survival analysis found that the RIS4 group had a poor prognosis (P <0.001). **(E)** Construction of the nomogram. Prognostic **(F)** The AUC predicted by Nomogram for patient prognosis remains around 0.8, which is significantly higher than other clinical features. **(G)** The benefit rate of patients receiving timely clinical treatment based on Nomogram is higher than other clinical features.

Subsequently, we performed a meta-analysis of the prognostic impact of NCPTS in different cohorts. In **Figure 8A**, pooled HR values were found to show that NCPTS remained a contributing factor to poor patient outcome. To further facilitate risk group classification and management of patients with clinical pancreatic cancer, we performed decision tree analysis on NCPTS and clinical characteristics. As shown in **Figure 8B**, patients can be divided into 4 risk groups RIS1, RIS2, RIS3 and RIS4 according to the level of NCPTS, gender and N stage. As shown in **Figure 8C**, RIS4 patients were all distributed in the NCPTS_high group, while RIS1, RIS2, and RIS3 were distributed in the NCPTS_low group. Survival analysis found that the RIS4 group had a poor prognosis (P <0.001, **Figure 8D**). Finally, a nomogram is constructed, as shown in **Figure 8E**, and the 1, 3 and 5 year mortality of TCGA-2J-AABK patients is 18.5%, 62.5% and 75.2%, respectively. Prognostic ROC curve analysis and decision curve analysis further evaluated the value of Nomogram. As shown in **Figure 8F**, we find that the AUC predicted by Nomogram for patient prognosis remains around 0.8, which is significantly higher than other clinical features. As shown in **Figure 8G**, the benefit rate of patients receiving timely clinical treatment based on Nomogram is higher than other clinical features.

### The role of the key gene EPS8 in pancreatic cancer cell lines was verified *in vitro*

**Figure 9:**
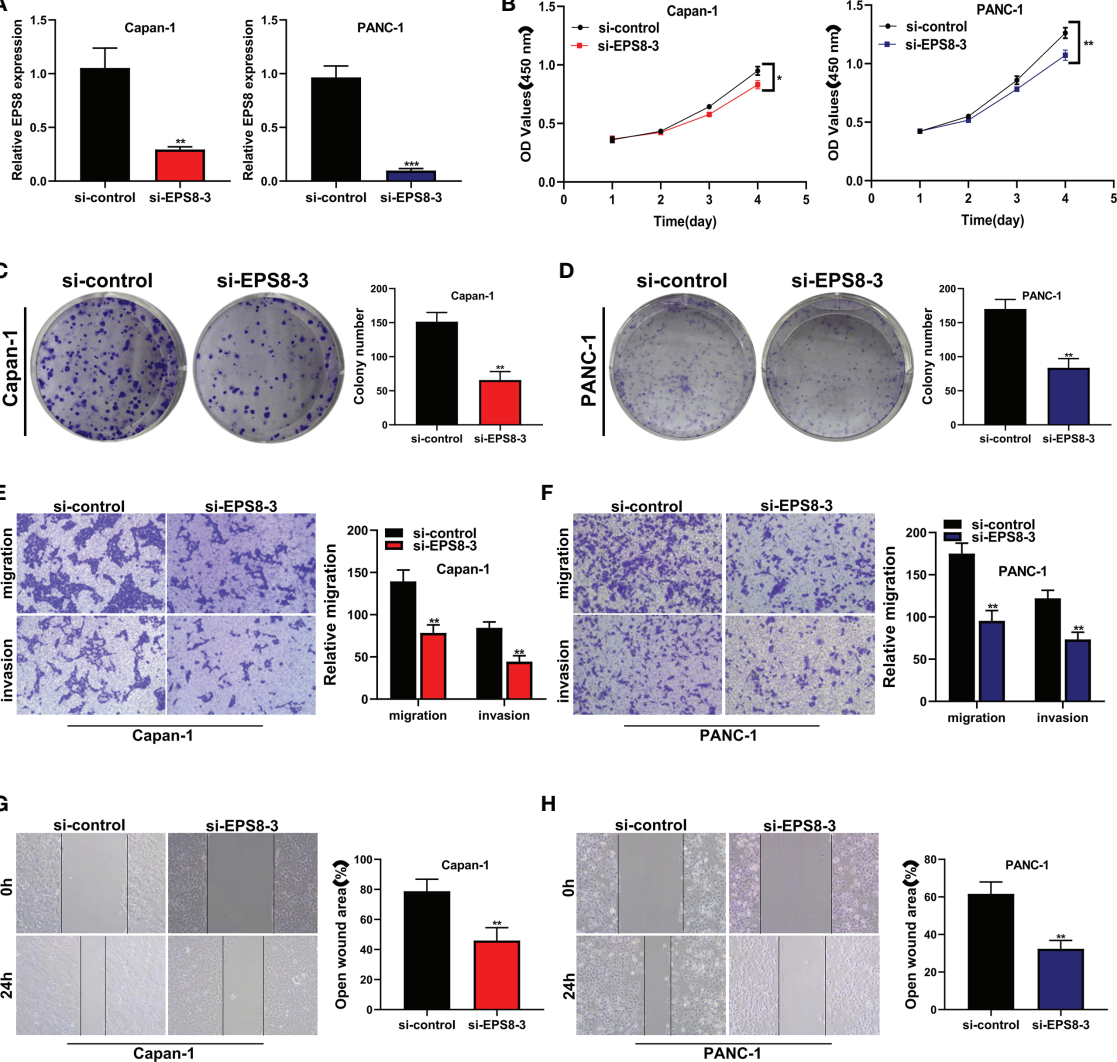
The role of the key gene EPS8 in pancreatic cancer cell lines was verified *in vitro*. **(A)** EPS8 was dramatically reduced In both the CAPAN-1 and PANC-1 cell lines (**P<0.01, ***P<0.001). **(B)** The activity of pancreatic cancer cells was dramatically reduced following EPS8 knockdown in CAPAN-1 and PANC-1 cell lines (*P<0.05, **P<0.01). **(C, D)** Clonal formation assays revealed that the ability of the CAPAN-1 cell line and the PANC-1 cell line to produce colonies was considerably reduced following EPS8 knockdown (**P<0.01). **(E, F)** EPS8 knockdown dramatically reduced the migration and invasion capacity of pancreatic cancer cells in the CAPAN-1 cell line and PANC-1 cell line in the transwell experiment (**P<0.01). **(G, H)** The migration ability of EPS8 group was weaker in si-EPS8 group in the wound healing experiment in the CAPAN-1 cell line and PANC-1 cell line(**P<0.01).

Because siEPS8-3 showed the highest knockdown efficiency of the three siRNAs, it was chosen for further tests and analysis. In both the CAPAN-1 and PANC-1 cell lines, EPS8 was dramatically reduced (**Figure 9A**; **P<0.01, ***P<0.001). The activity of pancreatic cancer cells was dramatically reduced following EPS8 knockdown in CAPAN-1 and PANC-1 cell lines (**Figure 9B**; *P<0.05, **P<0.01). Following that, clonal formation assays revealed that the ability of the CAPAN-1 cell line (**Figure 9C**) and the PANC-1 cell line (**Figure 9D**) to produce colonies was considerably reduced following EPS8 knockdown (**P<0.01). EPS8 knockdown dramatically reduced the migration and invasion capacity of pancreatic cancer cells in the CAPAN-1 cell line and PANC-1 cell line (**Figures 9E, F**) in the transwell experiment (**P<0.01). The migration ability of EPS8 group was weaker in si-EPS8 group in the wound healing experiment in the CAPAN-1 cell line and PANC-1 cell line (**Figures 9G, H** **;** **P<0.01).

### The expression of EPS8 in pancreatic cancer tissues was verified by PCR assay of clinical samples

**Figure 10:**
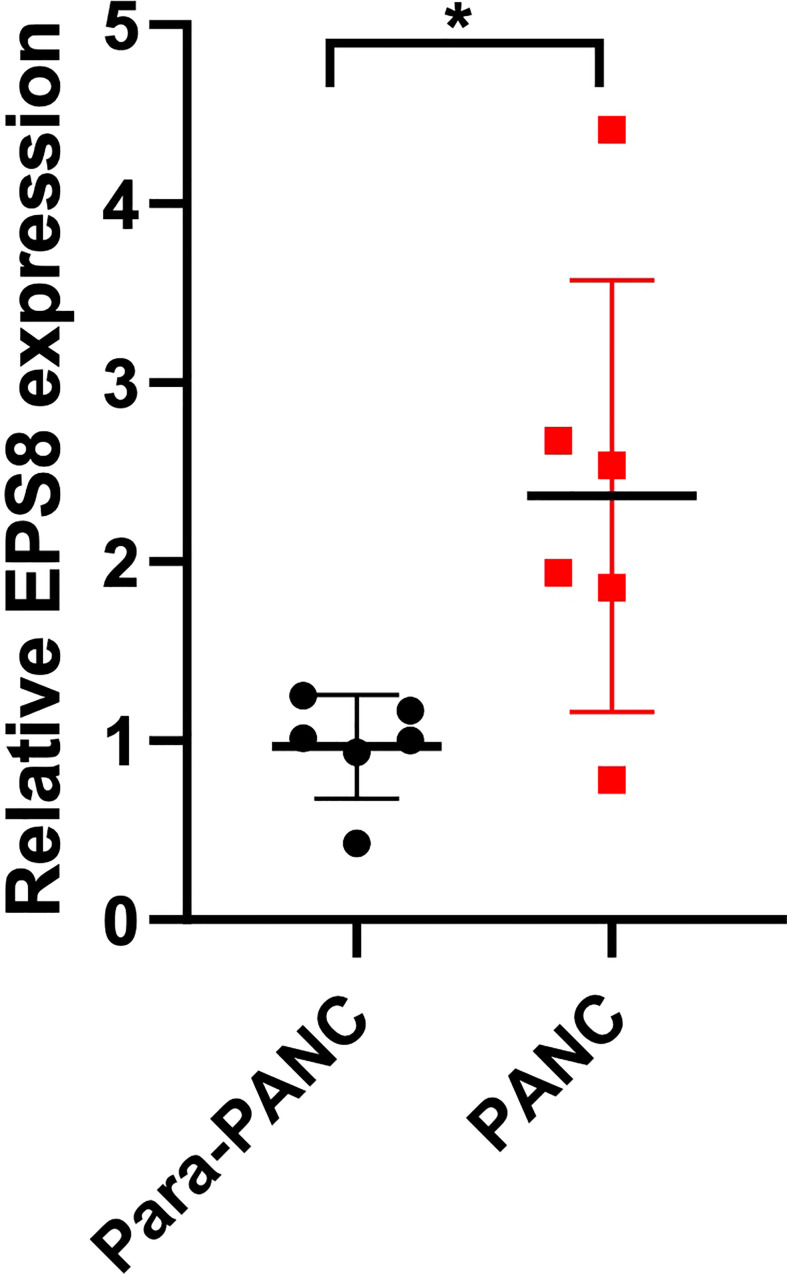
Clinical validation of EPS8 expression. The expression of EPS8 was significantly up-regulated in pancreatic cancer (*P<0.05).

To further validate EPS8 expression in clinical specimens, PCR assays were performed. It was found that compared with para-tumor tissue, the expression of EPS8 in pancreatic cancer was significantly up-regulated (**Figure 10**, *P<0.05).

## Discussion

Pancreatic cancer is considered to be a devastating tumor, with a very high mortality rate and a 5-year survival rate of less than 10%, although its incidence is low (19). The development of pancreatic cancer is a multi-step process involving changes in many endogenous and exogenous factors (20). Its complex tumor microenvironment and resistance to cell death result in rapid tumor progression and poor treatment (20). The current mechanism of programmed cell death is regarded as a promising treatment for tumors (21). Common programmed pathways of cell death include pyroptosis, ferroptosis, necroptosis, etc (22). They play a dual role in tumor development, and this complex crosstalk is currently a major challenge in understanding programmed cell death in tumors. Necroptosis is a newly defined type of programmed cell death whose role in pancreatic cancer remains unclear.

The role of necroptosis in pancreatic cancer has been tentatively discussed. Some studies have identified necroptosis as a progenitor of pancreatic cancer, while others have identified necroptosis as a suppressor and a promising future treatment for pancreatic cancer. Seifert et al. found that necrosome promotes pancreatic cancer progression through CXCL1-mediated immunosuppression (23). Ando et al. found that CXCL5 released by pancreatic cancer cells during necroptosis promotes cancer cell migration and invasion (24). However, Xie et al. found that aurora kinase inhibitor CCT137690 slowed the growth of pancreatic ductal adenocarcinoma cells by inducing necroptosis (25). Zhao et al. found that pyridazinone compound IMB5036 inhibited the proliferation of pancreatic cancer cells by necroptosis activation (26). Thus, necroptosis’s role in pancreatic cancer is complex and two-sided. Whether it can become a powerful weapon in the treatment of pancreatic cancer needs to be explored in depth.

In this study, we explored the significance of necroptosis in pancreatic cancer through bioinformatics analysis. First, we identified necroptosis genes using weighted co-expression network analysis (WGCNA) and single-cell sequencing. The prognostic signature of necroptosis was constructed by COX and Lasso regression for these necroptosis genes. The signature is made up of eight genes, Its calculation formula is NCPTS = POLR3GL * (-0.404) + COL17A1 * (0.092) + DDIT4 * (0.007) + PDE4C * (0.057) + CLDN1 * 0.075 + HMGA2 * 0.056 + CENPF * 0.198 +EPS8 * 0.219. With signature, we were able to group patients at risk, with a significantly poorer prognosis in the high-risk group. This is undoubtedly beneficial to the prognosis assessment of patients with pancreatic cancer. In addition, there were differences in predicted immune infiltration level, immunotherapy response, and tumor mutation load among different groups, which provided reference for the exploration of tumor microenvironment in pancreatic cancer.

At present, the application of immunotherapy in tumor is developing rapidly, and has achieved preliminary results in many solid tumors or hematological tumors (27). However, conventional immunotherapy regimensuch as PD-1/PD-L1 receptor inhibitors have shown limited efficacy in pancreatic cancer (28). Only a small percentage of pancreatic cancer patients respond well to immunotherapy. Single drug immunotherapy is no longer sufficient to treat pancreatic cancer (29). Many studies have looked at combining immunotherapy with other treatments such as chemotherapy, radiotherapy, and targeted therapies to better benefit patients with pancreatic cancer (30). Among them, the combination of therapy based on programmed cell death (pyroptosis, ferroptosis, necroptosis) and immunotherapy is a promising direction (31). Our study examined necroptosis and the immune microenvironment in pancreatic cancer. The results showed that there were significant differences in immune microenvironment among groups based on signature, including immune score, level of immune cell infiltration, expression level of immune checkpoint genes, and tumor mutation load. This has implications for immunotherapy and necroptosis based therapies for pancreatic cancer.

Wu et al. constructed a necroptosis-related prognostic signature composed of 10 genes, including MET, CASKIN2, TLE2, USP20, MROH9, SPRN, ARSG, ARNTL2, ANLN, and LY6D (32). They found that increased risk scores were associated with increased mortality. Moreover, different risk groups had different levels of pathway enrichment and immune cell infiltration. Ma et al. performed pan-cancer analysis of necroptosis related genes and found that necroptosis predicted prognosis and immune status in patients with pancreatic adenocarcinoma (PAAD) (33). Ding et al. constructed a prognostic signature associated with necroptosis in pancreatic cancer by differential expression analysis and regression analysis, in which patients with risk scores may be correlated with chemotherapy sensitivity (34). Shi et al. found that GLUD1, SPATA2, H2AC8, PYGL and TNFS10 among the genes related to necroptosis may be closely related to the prognosis of pancreatic cancer patients, and may be potentially related to the activation of PI3K/AKT pathway (35). In contrast, our study is the first analysis of necroptosis associated genes in pancreatic cancer combined with a single cell sequencing dataset. We also used more external validation sets to verify the results, such as PA-CA cohort, PA-AU Cohort, GSE85916 cohort. Most importantly, our study validated the results using cell lines and clinical samples and identified EPS8 as a novel marker for pancreatic cancer. However, our study also has some limitations. We lack mechanistic experiments and relevant animal experiments to verify the regulation mechanism of necroptosis gene in pancreatic cancer, which will be further explored in the future.

## Conclusion

We constructed a necroptosis-related prognostic signature for pancreatic cancer by combining the results of single-cell sequencing and transcriptome analysis. Our signature can effectively evaluate the prognosis of patients with pancreatic cancer and provide reference for the treatment of pancreatic cancer to a certain extent.

## Data availability statement

The original contributions presented in the study are included in the article/Supplementary Material. Further inquiries can be directed to the corresponding authors.

## Ethics statement

The studies involving human participants were reviewed and approved by Fuyang Hospital Affiliated to Anhui Medical University. The patients/participants provided their written informed consent to participate in this study.

## Author contributions

LC designed the study. LC, TZ, ZX were involved in database search and statistical analyses. LC and WW were involved in the writing of manuscript and its critical revision. HY and HZ was responsible for the submission of the final version of the paper. All authors contributed to the article and approved the submitted version.

## Funding

This study was supported by the Foundation of Jiaxing Science and Technology Bureau (2021AD30140).

## Acknowledgments

We are very grateful for data provided by databases such as TCGA, GEO.

## Conflict of interest

The authors declare that the research was conducted in the absence of any commercial or financial relationships that could be construed as a potential conflict of interest.

## Publisher’s note

All claims expressed in this article are solely those of the authors and do not necessarily represent those of their affiliated organizations, or those of the publisher, the editors and the reviewers. Any product that may be evaluated in this article, or claim that may be made by its manufacturer, is not guaranteed or endorsed by the publisher.
